# Cannabidiol and Amisulpride Improve Cognition in Acute Schizophrenia in an Explorative, Double-Blind, Active-Controlled, Randomized Clinical Trial

**DOI:** 10.3389/fphar.2021.614811

**Published:** 2021-04-29

**Authors:** F. Markus Leweke, Cathrin Rohleder, Christoph W. Gerth, Martin Hellmich, Ralf Pukrop, Dagmar Koethe

**Affiliations:** ^1^ Department of Psychiatry and Psychotherapy, Medical Faculty Mannheim, Central Institute of Mental Health, Heidelberg University, Mannheim, Germany; ^2^ Youth Mental Health Team, Brain and Mind Centre, Faculty of Medicine and Health, Central Clinical School, The University of Sydney, Sydney, NSW, Australia; ^3^ Department of Psychiatry and Psychotherapy, Faculty of Medicine and University Hospital Cologne, University of Cologne, Cologne, Germany; ^4^ Department of General Psychiatry, Rheinhessen-Fachklinik Alzey, Alzey, Germany; ^5^ Institute of Medical Statistics and Computational Biology, Faculty of Medicine and University Hospital Cologne, University of Cologne, Cologne, Germany

**Keywords:** cognition, neuropsychological function, cannabidiol, schizophrenia, endocannabinoids, RCT, human

## Abstract

Cannabidiol (CBD), a principal phytocannabinoid constituent, has demonstrated antipsychotic properties in recent clinical trials. While it has also been suggested a promising candidate for the treatment of neurodegenerative disorders, it failed to demonstrate efficacy in cognitive impairments associated with schizophrenia as an add-on treatment (600 mg/day for 6 weeks) in 18 chronically ill patients co-treated with a variety of psychopharmacologic drugs. Here, we report on the results of parallel-group, active-controlled, mono-therapeutic, double-blind, randomized clinical trial (CBD-CT1; ClinicalTrials.gov identifier: NCT00628290) in 42 acute paranoid schizophrenic patients receiving either CBD (up to 800 mg/day) or amisulpride (AMI, up to 800 mg/day) for four weeks in an inpatient setting with neurocognition as a secondary objective. Twentynine patients (15 and 14 in the CBD and AMI group, respectively) completed two cognitive assessments at baseline and the end of the treatment period. We investigated the following cognitive domains: pattern recognition, attention, working memory, verbal and visual memory and learning, processing speed, and verbal executive functions. When applying the Bonferroni correction for multiple testing, *p* < 0.0004 would indicate statistical significance. There was no relevant difference in neurocognitive performance between the CBD and the AMI group at baseline, and we observed no post-treatment differences between both groups. However, we observed improvements within both groups from pre-to post-treatment (standardized differences reported as Cohen’s *d*) in visual memory (CBD: 0.49, *p* = 0.015 vs. AMI: 0.63, *p* = 0.018) and processing speed (CBD: 0.41, *p* = 0.004 vs. AMI: 0.57, *p* = 0.023). Furthermore, CBD improved sustained attention (CBD: 0.47, *p* = 0.013, vs. AMI: 0.52, *p* = 0.085), and visuomotor coordination (CBD: 0.32, *p* = 0.010 vs. AMI: 0.63, *p* = 0.088) while AMI led to enhanced working memory performance in two different paradigms (Subject Ordered Pointing Task–AMI: 0.53, *p* = 0.043 vs. CBD: 0.03, *p* = 0.932 and Letter Number Sequencing–AMI: 0.67, *p* = 0.017 vs. CBD: 0.08 *p* = 0.755). There was no relevant correlation between changes in neurocognitive parameters and psychotic symptoms or anandamide serum levels. This study shows that both CBD and AMI improve neurocognitive functioning with comparable efficacy in young and acutely ill schizophrenia patients via an anandamide-independent mechanism.

## Introduction

Schizophrenia is a complex psychiatric syndrome including positive symptoms (delusions, hallucinations, thought disorder) and negative symptoms (anhedonia, blunted affect, social withdrawal) as well as cognitive impairment (American Psychiatric Association, 2013). The disease concept has been originally described by Emil Kraepelin as “dementia praecox” or premature dementia. Originally, dementia was synonymous with insanity and not related to age, cognitive status, or reversibility (Adityanjee et al., 1999). In contrast, Kraepelin used it in the more modern sense of the word, including the cognitive decline he observed. Eugen Bleuler pointed to a temporary remission and even recovery of the “dementia praecox” syndrome he named “schizophrenia” (Bleuler, 1908). Nevertheless, more than a century later, the cognitive impairment in a large number of patients remains among the most difficult to influence aspects of schizophrenia.

The fundamental dimensions of cognitive deficits in schizophrenia encompass memory, attention, working memory, problem-solving, processing speed, and social cognition (Nuechterlein et al., 2004; Keefe and Fenton, 2007). Despite a considerable heterogeneity in cognitive symptoms, approximately 65–80% of patients show clinically significant impairments and perform one to two standard deviations (SD) below the population mean (Kremen et al., 2000; Keefe and Fenton, 2007; Mesholam-Gately et al., 2009; Uren et al., 2017; Islam et al., 2018; McCleery and Nuechterlein, 2019). However, it has been suggested that even those individuals who had been rated to perform as “within normal limits” exhibit a cognitive decline compared with what their cognitive functions would have been if they had never developed the illness (Kremen et al., 2000; Keefe and Fenton, 2007; Vaskinn et al., 2014). Another study reported that even patients with overall normal cognitive and intellectual functioning showed impairments in processing speed-dependent domains (Bechi et al., 2019).

Cognitive deficits are already profound early in the course of the illness (Riley et al., 2000; Mesholam-Gately et al., 2009), only modestly related to negative symptoms, essentially independent of positive symptom severity (Keefe et al., 2006), and exist already prior to the initiation of antipsychotic treatment (Saykin et al., 1994), indicating that they are not merely the result of other schizophrenia symptoms or psychotropic treatments. Furthermore, the presence of cognitive deficits or cognitive decline during adolescence has been found to predict the conversion to schizophrenia (Cannon et al., 2002; Reichenberg et al., 2005; Keefe et al., 2006; Keefe and Fenton, 2007; Seidman et al., 2016; Lam et al., 2018), supporting the view that significant cognitive deficits precede the onset of psychotic symptoms (McCleery and Nuechterlein, 2019).

The various cognitive deficits have been shown to contribute to functional outcomes, such as interpersonal relationships, social problem-solving, participation in recreational and community activities, occupational and vocational functioning, and self-care (Green et al., 2000; Bryson and Bell, 2003; Mohamed et al., 2008; Fervaha et al., 2014; Chang et al., 2016). Consequently, cognition is an important treatment target. Currently available antipsychotic medications can yield modest beneficial effects on cognitive functioning, although the findings have been inconsistent regarding whether atypical antipsychotics confer greater effects than typical antipsychotics (Woodward et al., 2005; Keefe et al., 2007; McCleery and Nuechterlein, 2019). Notably, detrimental effects of antipsychotics on cognition are also possible. This has been associated with very high dopamine D_2_ receptor occupancy level, very high dosing, polypharmacy, and concomitant use of anticholinergic medications (Hori et al., 2006; Sakurai et al., 2013; McCleery and Nuechterlein, 2019).

Due to the lack of marked cognitive benefits of standard antipsychotics, alternative pharmacological treatments with different mechanisms of action are currently investigated, including cholinergic agents, dopamine D_1_ agonists, and glutamatergic agents (Buchanan et al., 2007).

Another promising novel antipsychotic agent is cannabidiol (CBD), a major ingredient of *Cannabis sativa*. CBD has demonstrated antipsychotic properties in small case studies (Zuardi et al., 1995; Zuardi et al., 2006; Zuardi et al., 2009; Makiol and Kluge, 2019) and randomized, placebo-controlled clinical trials in acutely and non-acutely ill schizophrenia patients (Leweke et al., 2012; McGuire et al., 2018). Moreover, first placebo-controlled, double-blind studies showed that CBD might also have beneficial effects in individuals in a Clinical-High-Risk (CHR) mental state for psychosis (Bhattacharyya et al., 2018; Appiah-Kusi et al., 2020).

However, while CBD has also been suggested to be a promising therapeutic candidate for treating neurodegenerative diseases through multifaceted molecular mechanisms (Cassano et al., 2020), it failed to demonstrate efficacy in ameliorating cognitive impairments in schizophrenia patients. In a small double-blind, placebo-controlled, three-parallel-arm clinical trial with non-acutely ill schizophrenia patients, a single dose of 300 (N = 9) or 600 mg (N = 9) CBD did not improve cognitive performance–more precisely selective attention–compared to placebo (N = 10) (Hallak et al., 2010). Furthermore, in comparison to placebo (N = 18), a six-week treatment with CBD (600 mg/day; N = 18) did not improve the cognitive performance (assessed with the MATRICS Consensus Cognitive Battery) of stable antipsychotic-treated patients diagnosed with chronic schizophrenia (Boggs et al., 2018). At the same time, a double-blind, randomized, placebo-controlled, parallel-group clinical trial investigating the efficacy of a higher CBD dosage (1000 mg/day, over eight weeks) as an add-on to stable antipsychotic medication in sub-acute schizophrenia spectrum patients (*n* = 43), observed a slightly improved cognitive performance (Brief Assessment of Cognition in Schizophrenia (BACS) composite score and subdomain “executive functions”) compared to those who received placebo (*n* = 45) as a secondary outcome. Although these differences did not reach statistical significance, the motor speed improvements were significantly greater in the CBD than in the placebo group (McGuire et al., 2018).

In comparison to these previously published studies, the present study compared the effects of a mono-therapeutic approach with CBD or the second-generation antipsychotic AMI in earlier stages of schizophrenia on six neurocognitive domains (pattern recognition; sustained attention; working memory; verbal and visual memory and learning; processing speed; verbal executive functions) in 42 acute paranoid schizophrenic patients as a secondary objective. AMI has been chosen as a comparator because of its clear dopamine D_2/3_-receptor antagonistic mechanism of action. In contrast, CBD’s antipsychotic potential has been found to be substantially linked to an increase in anandamide levels (Leweke et al., 2012).

## Methods

### Subjects

This therapeutic-exploratory (phase II), double-blinded, monocenter, randomized, parallel-group, controlled clinical trial (RCT) of CBD vs. AMI (CBD-CT1; ClinicalTrials.gov. Identifier: NCT00628290) was approved by the Ethics Committee of the University of Cologne and the BfArM (Federal Institute for Drugs and Medical Devices). Initially, an independent psychiatrist assessed patients to confirm their ability to provide informed consent. After a detailed explanation of study procedures, written informed consent was obtained from each patient.

**FIGURE 1:**
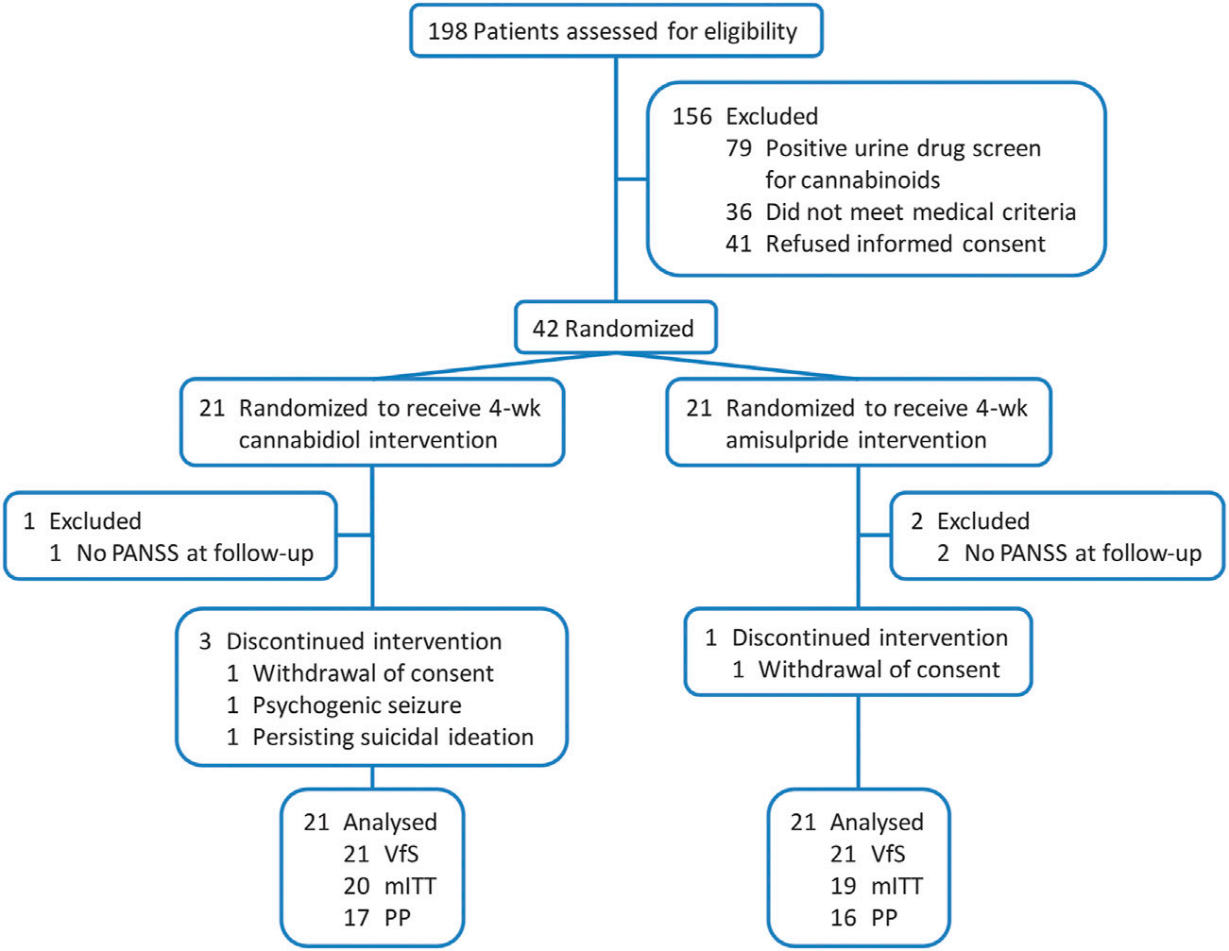
CONSORT flow diagram (Leweke et al., 2012). Analyses sets: VfS, valid for safety; mITT, modified intention-to-treat and PP, per protocol.

Details on the patient samples have been previously provided elsewhere (Leweke et al., 2012) and are summarized in Table 1. In brief, men and women aged 18–50 years and diagnosed with schizophrenia or schizophreniform psychosis, with a total Brief Psychiatric Rating Scale (BPRS) score ≥36 and a BPRS thought disorder score ≥12, were eligible to participate in the study. Exclusion criteria comprised a positive urine drug screening for illicit drugs, other relevant psychiatric disorders, treatment with a depot antipsychotic within three months before participation in the study, history of treatment resistance, a relevant and/or unstable medical condition, and insufficient contraception, pregnancy, or breast-feeding in female patients. The consort diagram (Figure 1) demonstrates the participants’ flow. Out of 42 inpatients with acute paranoid schizophrenia, 33 completed the study per protocol (participation in the study throughout the planned course). After a screening period of up to 7 days and a minimum period of three antipsychotic-free days (the vast majority of patients was antipsychotic-naïve or hospitalized for acute exacerbation after discontinuing antipsychotic treatment and therefore off antipsychotic well before inclusion in our study), patients were randomized (1:1) to receive either CBD or AMI starting with 200 mg per day each and increased stepwise by 200 mg per day to a daily dose of 200 mg four times daily (total 800 mg per day) each within the first week that was maintained for another three weeks. A reduction of each treatment to 600 mg per day was allowed for clinical reasons, such as unwanted side effects after week 2, which was the case for three patients in the CBD and five patients in the AMI arm. The assessment of the change of neurocognitive performance was a secondary objective of this RCT. Fifteen patients of the CBD and 14 patients of the AMI group were able to complete neuropsychological assessments prior to initiation of treatment (day -8 to -1) and on the last day of active treatment with either CBD or AMI (day 28). However, only 12 and 11 patients treated with CBD and AMI respectively completed all neurocognitive tests.

**TABLE 1:** Characteristics of the patient sample.

	Full analyis set			Subset with full neurocognitive assessment		
	CBD (n = 20)	AMI (n = 19)	CBD vs. AMI P-Value^a^	CBD (n = 15)	AMI (n = 14)	CBD vs. AMI P-Value^a^
Demographic characteristics						
Age, years (mean ± SD)	29.7 ± 8.3	30.6 ± 9.4	0.966	28.8 ± 7.7	30.3 ± 9.7	0.844
Male gender, count (%)	15 (75.0)	17 (89.5)	0.407	12 (80.0)	13 (92.9)	0.598
Baseline severity of illness scores, mean ± SD						
PANSS Total	91.2 ± 14.0	99.5 ± 17.1	0.736	89.9 ± 15.9	98.9 ± 16.7	0.347
PANSS Positive	24.6 ± 5.6	22.5 ± 6.2	0.205	23.5 ± 5.3	23.4 ± 6.3	0.678
PANSS Negative	23.7 ± 5.4	25.3 ± 5.6	0.573	23.8 ± 5.8	25.7 ± 6.1	0.511
PANSS General	42.9 ± 8.6	47.7 ± 11.4	0.155	42.5 ± 9.4	49.9 ± 11.1	0.063
BPRS	58.1 ± 9.7	57.7 ± 10.3	0.764	56.1 ± 9.8	59.4 ± 8.9	0.541
CGI	6.3 ± 0.7	6.8 ± 0.4	**0.011**	6.3 ± 0.6	6.7 ± 0.5	**0.037**
Other, mean ± SD						
Lorazepam, mg/day	2.2 ± 1.6	4.2 ± 2.4	**0.006**	2.0 ± 1.7	3.8 ± 2.3	0.055
SAS	36.4 ± 7.7	36.9 ± 8.1	0.800	35.7 ± 6.8	37.6 ± 8.5	0.584
EPS	0.1 ± 0.2	0.0 ± 0.1	0.485	0.1 ± 0.2	0.0 ± 0.0	0.682
Changes in PANSS after 28 days of treatment, mean ± SD; n [all changes significant compared to baseline, p < 0.001 in the full analysis set Leweke et al. (2012)]						
PANSS Total	−30.5 ± 16.4; 17	−30.1 ± 24.7; 18	0.843	−31.3 ± 16.8	−37.0 ± 21.4	0.332
PANSS Positive	−9.0 ± 6.1; 17	−8.4 ± 7.5; 18	0.519	−8.9 ± 6.3	−10.1 ± 7.3	0.903
PANSS Negative	−9.1 ± 4.9; 17	−6.4 ± 6.0; 18	0.234	−9.6 ± 5.1	−7.9 ± 5.7	0.527
PANSS General	−12.5 ± 10.4, 17	−15.3 ± 14.3; 18	0.457	−12.8 ± 10.1	−19.0 ± 13.0	0.159

CBD, cannabidiol; AMI, amisulpride; BPRS, brief psychiatric rating scale; CGI, clinical global impression scale; EPS, extrapyramidal symptoms rating scale; PANSS, positive and negative syndrome scale; SAS, social anxiety scale. Full analysis set (Leweke et al., 2012) and subset of patients who completed neuropsychological assessments prior to initiation of treatment. Please note, the *p*-values for changes in PANSS slightly differ from the ones given in the main article (Leweke et al., 2012), since therein results based on a mixed model for repeated measures with baseline adjustment are reported. However, both approaches support the same conclusions.

a The Kruskal-Wallis test (continuous data) or Fisher’s exact (nominal data) test. Statistical significance between groups (p ≤ 0.05) is indicated in bold.

### Neurocognitive Assessment

The neurocognitive test battery took approximately 2 h to complete and was conducted by fully qualified neuropsychologists. Furthermore, training effects were diminished by using parallel versions of each test at t_0_ and t_1_. Premorbid verbal IQ was tested with the German version of the Multiple Choice Vocabulary Test (Lehrl, 1995), which is highly correlated with total IQ.

The test battery comprised 10 validated neuropsychological tests with good test-retest reliability (Delaney et al., 1992; Keefe et al., 2004; Ross et al., 2007; Nuechterlein et al., 2008) to assess the following neurocognitive domains:

(1) Pattern recognition. A computerized version of a *visual backward masking task* with letters F, H, or T as target stimuli and one of four masking conditions [random dot pattern or letter pattern masking stimulus after short (42 ms) or long (104 ms) interstimulus intervals] provided a measure of visual information-processing (number of hits). The session consisted of three blocks of 30 trials, each including six trials of each masking condition and six no-mask control trials presented in random order. To enhance reliability for the measurement of pattern recognition, aggregated scores across the different masking conditions were calculated.
(2) Attention. The *Continuous Performance Test* (identical pairs version (Cornblatt, 1996)) provided a measure of sustained attention. The signal detection parameter d’ was calculated across 150 trials with 4-digits stimuli (emphasis on left hemispheric processing) and 150 trials with meaningless symbols stimuli (emphasis on right-sided processing).
(3) Working memory. The *Letter Number Sequencing* (Gold et al., 1997) requires subjects to sort letters from numbers within a sequence of alternating letters and numbers read to them and recall the letters and numbers in ascending orders separately. During each trial of a computerized version of the *Subject Ordered Pointing Task* (Petrides and Milner, 1982), subjects had to point to 1 of 12 objects, and the relative positions of the objects varied randomly across trials. Across three sessions of 12 trials, the number of errors (pointing to an object already chosen on a previous trial) was calculated. During each trial of the *Delayed Response Task* (Park and Holzman, 1992; Pukrop et al., 2003) for spatial working memory, a black dot was presented for 200 ms at 1 of 16 possible positions of a circle followed by a specific delay (5 s or 15 s). During the delay period, subjects had to solve arithmetic distractor tasks. After the delay period, subjects were required to indicate the position of the previously presented dot on a touch-sensitive monitor to determine the Euclidean distance to the target. To enhance reliability for the measurement of the spatial working memory, aggregated scores across the two different delay periods were calculated.
(4) Verbal and visual memory and learning. The *Auditory Verbal Learning Test* (Lezak et al., 2012) provided measures for the immediate recall capabilities after one to five learning trials of word lists and delayed recall performance. A measure of visual memory was provided by the *Rey-Osterrieth Complex Figure Test* (Rey, 1941), calculating the delayed recall performance by a standardized scoring procedure.
(5) Processing speed. The *Digit-Symbol-Test* (Kaplan et al., 1991) and *Trail-Making Test A and B* (Reitan and Wolfson, 1985) provided measures for the speed of visual information-processing and visuomotor coordination. The ratio of TMT-B to TMT-A can be interpreted as an indicator of the cognitive component independent of the motor component.
(6) Verbal executive functions. A *verbal fluency task* (sum of four lexical and semantic category tasks) was used to measure verbal executive functions (Turek et al., 2017; Palsetia et al., 2018).

These domains of cognitive functioning are those found to be consistently impaired and related to outcome in schizophrenia and are assessed by the BACS as well (Keefe et al., 2004) that was not available at the time the trial started.

### Determination of Anandamide Levels

For a subgroup of patients, serum anandamide levels were determined before (baseline) and after CBD (N = 14) or AMI (N = 8) treatment (day 28) by liquid chromatography/electrospray tandem mass spectrometry (LC/ESI-MS-MS) using a method following international guidelines and requirements for the validation of a method and the quantitative evaluation of the compounds as described previously in detail (Giuffrida et al., 2000; Schreiber et al., 2007; Leweke et al., 2012).

### Statistical Analysis

Since observed distributions of all neuropsychological characteristics could be well approximated by normal distributions, parametric methods (from the t-test family) were used to evaluate differences in location. Due to the explorative character of the study, the type I error (alpha) was not adjusted for multiplicity. Thus, the results need to be interpreted carefully. When applying the Bonferroni correction, *p* < 0.0004 (i.e., 0.05/120) would indicate statistical significance. Throughout the manuscript, we added the Bonferroni-corrected *p*-values (p_corr_) in square brackets. Alternatively, also based on given *p* values, readers may prefer to apply the Benjamini-Hochberg method (Benjamini and Hochberg, 1995) to control the false discovery rate: The *p* values need to be ranked; find the maximum rank k with p_(k)_ ≤i/120*0.05; reject all null hypotheses with *p* values of rank i≤k.

Standardized differences for neuropsychological performance changes from t0 to t1 in both groups are reported as Cohen’s d (within groups over time, i.e., standardized mean gain (t_0_ minus t_1_), and between groups, i.e., standardized mean difference (AMI minus CBD), respectively) (Lipsey and Wilson, 2001). Thus, in both cases, Cohen’s d was calculated based on the pooled standard deviation (either pooled over time or pooled over groups). Associations between neuropsychological performance and psychopathological symptoms were described by Pearson’s correlation coefficient. Moreover, for a subgroup of patients, serum anandamide levels were available (Leweke et al., 2012). Thus, associations between changes of the neurocognitive performance and serum anandamide levels were assessed by a median slope analysis (Wilcoxon signed-rank test of the null hypothesis that the distribution of slopes is symmetric about zero). This type of analysis is consistent with our previous analysis to assess the association of the change of anandamide levels in serum and the change in the PANSS total score (Leweke et al., 2012). Calculations were done with the software SPSS Statistics (IBM Corp., Armonk, NY, United States) and Stata/SE (StataCorp LLC, College Station, TX, United States).

## Results

**FIGURE 2:**
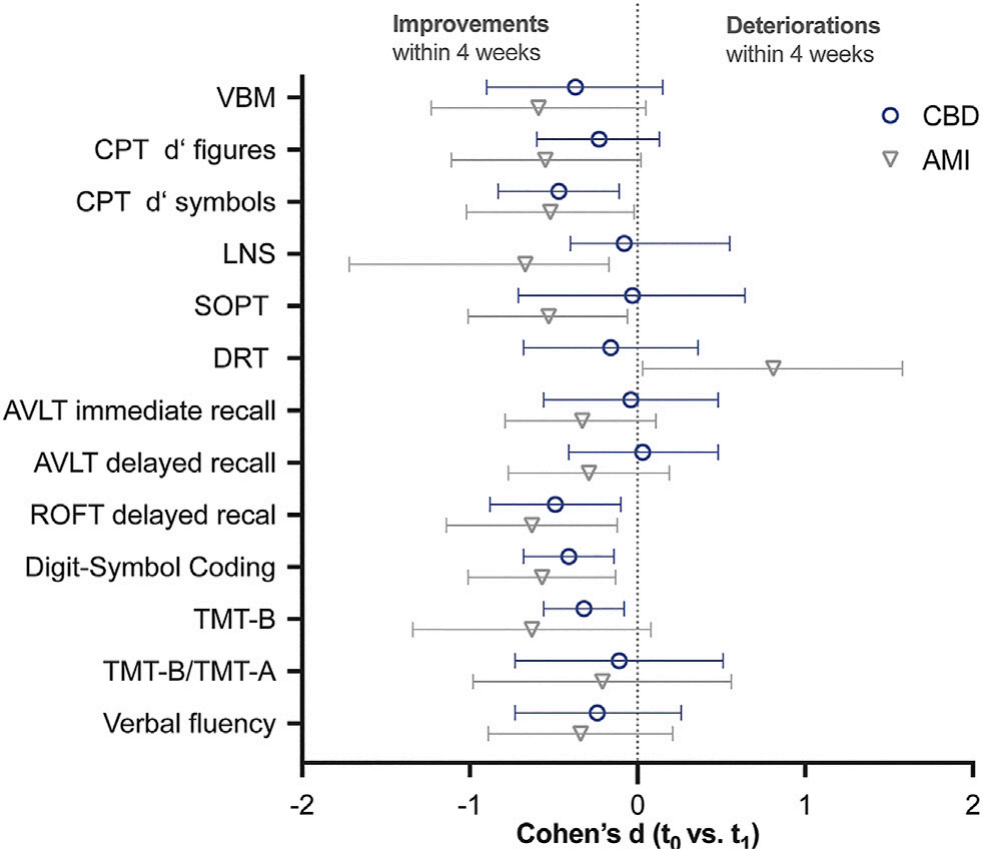
Effect size [Cohen’s d (95% CI)] for changes in neurocognitive test scores (t_0_-t_1_ mean). Cohen’s d for SOPT (#error), DRT (Euclidian distance), TMT-B, and ratio TMT-B/TMT-A (both times in s) have been inverted to allow for a better comparison. Thus, improvements of neurocognitive functioning within four weeks of treatment are indicated by negative effect sizes. 95% CI that do not contain 0, indicate significant improvements at the 5% level. CBD, cannabidiol; AMI, amisulpride; AVLT, auditory verbal learning test; d′, signal detection parameter; DRT, delayed response task, LNS, letter number sequencing; ROFT, Rey-Osterrieth complex figure test; SOPT, subject ordered pointing task; TMT-A, trail-making test A; TMT-B, trail-making test B; VBM, visual backward masking.

Both treatment groups improved on all neuropsychological functions from pre-to post-treatment (except for slight non-significant deteriorations of verbal working memory performance assessed by AVLT–delayed recall (*p* = 0.881 [p_corr_ = 1], d = -0.03) and the Letter Number Sequencing (*p* = 0.366 [p_corr_ = 1], d = 0.08) in the CBD group, and of working memory performance assessed by the delayed response task in the AMI group (*p* = 0.066 [p_corr_ = 1], d = -0.81); Table 2). In the AMI group, t-tests (Table 2) revealed improvements from t0 to t1 on the Letter Number Sequencing Test and Subject Ordered Point Task (both working memory tests), as well as the Rey-Osterrieth Complex Figure Test (ROFT, visual memory) and the Digit-Symbol Test (processing speed). Patients treated with CBD showed improvements on the Continuous Performance Test–symbol stimuli (sustained attention), the ROFT (visual memory), Digit-Symbol Test (processing speed), and Trail-Making Test B (visuomotor coordination). Effect sizes (Table 3'; Figure 2) for all improvements ranged from 0.21 (visuomotor coordination) to 0.67 (verbal working memory) in the AMI group and from 0.03 (working memory) to 0.49 (visual memory) in the CBD group. We did not find significant differences between treatment groups.

**TABLE 2:** Changes in neurocognitive performance (raw score means ± SD) before (t_0_) and after (t_1_) intervention. Statistical significant changes (p ≤ 0.05) are indicated in bold.

	AMI			CBD		
	t_0_ (mean ± SD; N)	t_1_ (mean ± SD; N)	paired t-test (t_0_-t_1_), [95% CI]	t_0_ (mean ± SD; N)	t_1_ (mean ± SD; N)	paired t-test (t_0_-t_1_) [95% CI]
VBM %Hits	71.79 ± 17.20; 14	80.34 ± 12.27; 14	t_(13)_ = −1.75, *p* = 0.105 [*p*_corr_ = 1]	70.57 ± 16.79; 15	77.15 ± 18.35; 15	t_(14)_ = −1.45, *p* = 0.170 [*p*_corr_ = 1]
			[−19.14, 2.04]			[−16.35, 3.19]
CPT d’ figures	1.04 ± 0.55; 13	1.32 ± 0.68; 13	t_(12)_ = −1.68, *p* = 0.119 [*p*_corr_ = 1]	1.49 ± 1.08; 15	1.73 ± 0.79; 15	t_(14)_ = −1.31, *p* = 0.213 [*p*_corr_ = 1]
			[−0.65, 0.09]			[−0.63, 0.152]
CPT d’ symbols	1.31 ± 0.84; 13	1.68 ± 0.75; 13	t_(12)_ = −1.88, *p* = 0.085 [*p*_corr_ = 1]	1.91 ± 0.82; 15	2.30 ± 0.85; 15	t_(14)_ = −2.85, ***p* = 0.013** [*p*_corr_ = 1]
			[−0.80, 0.06]			**[**−**0.69,** −**0.10]**
LNS-# correct	14.36 ± 3.30; 11	16.36 ± 2.73; 11	t_(10)_ = −2.85, ***p* = 0.017** [*p*_corr_ = 1]	16.23 ± 3.11; 13	15.92 ± 4.43; 13	t_(12)_ = 0.32, *p* = 0.755 [*p*_corr_ = 1]
			**[**−**3.56,** −**0.44]**			[−1.80, 2.41]
SOPT # Errors	5.75 ± 2.83; 12	4.33 ± 2.15; 12	t_(11)_ = 2.28, ***p* = 0.043** [*p*_corr_ = 1]	4.58 ± 3.09; 12	4.50 ± 2.39; 12	t_(11)_ = 0.09, *p* = 0.932 [*p*_corr_ = 1]
			**[0.05, 2.78]**			[−2.02, 2.19]
DRT Euclidian distance	9.41 ± 18.76; 14	21.41 ± 11.08; 14	t_(13)_ = −2.00, *p* = 0.066 [*p*_corr_ = 1]	15.05 ± 23.47; 15	11.81 ± 16.06; 15	t_(14)_ = 0.60, *p* = 0.563 [*p*_corr_ = 1]
			[−24.93, 0.94]			[−8.49, 14.98]
AVLT immediate recall # correct	8.64 ± 3.63; 14	9.86 ± 4.11, 14	t_(13)_ = −1.30, *p* = 0.216 [*p*_corr_ = 1]	9.40 ± 3.54; 15	9.53 ± 3.09; 15	t_(14)_ = −0.15, *p* = 0.881 [*p*_corr_ = 1]
			[−3.23, 0.80]			[−2.02, 1.75]
AVLT delayed recall # Correct	8.07 ± 4.39; 14	9.29 ± 4.14; 14	t_(13)_ = −1.09, *p* = 0.296 [*p*_corr_ = 1]	8.80 ± 3.78; 15	8.67 ± 3.958; 15	t_(14)_ = 0.15, *p* = 0.881 [*p*_corr_ = 1]
			[−3.62, 1.20]			[−1.75, 2.02]
ROFT delayed recall standardized score	18.54 ± 7.19; 13	24.19 ± 8.83; 13	t_(12)_ = −2.74 ***p* = 0.018** [*p*_corr_ = 1] **[**−**10.16,** −**1.15]**	21.14 ± 7.17; 14	24.68 ± 7.17; 14	t_(13)_ = −2.80 ***p* = 0.015** [*p*_corr_ = 1]
						**[**−**6.27,** −**0.80]**

Digit-symbol coding # Correct	42.46 ± 9.37; 11	46.36 ± 7.41; 11	t_(10)_ = −2.68, ***p* = 0.023** [*p*_corr_ = 1]	52.15 ± 13.25; 13	57.46 ± 12.82; 13	t_(12)_ = −3.60, ***p* = 0.004** [*p*_corr_ = 0.480]
			**[**−**7.17,** −**0.65]**			**[**−**8.52,** −**2.10]**
TMT-B time in sec	120.73 ± 64.31; 11	83.64 ± 27.78; 11	t_(10)_ = 1.89, *p* = 0.088 [*p*_corr_ = 1] [−6.58, 80.76]	88.79 ± 49.70; 13	69.31 ± 32.24; 13	t(12) = 3.05, ***p* = 0.010** [*p*_corr_ = 1]
						**[5.55, 33.42]**
Ratio TMT-B/TMT-A time in sec	3.17 ± 1.19; 11	2.82 ± 1.38; 11	t_(10)_ = 0.65, *p* = 0.531 [*p*_corr_ = 1]	2.74 ± 0.94; 13	2.65 ± 0.61; 13	t_(12)_ = 0.36, *p* = 0.728 [*p*_corr_ = 1] [0.65, 0.36]
			[−0.84, 1.53]			
Verbal fluency	11.28 ± 4.16; 12	12.21 ± 2.63; 11	t_(10)_ = −0.92, *p* = 0.376 [*p*_corr_ = 1] [−3.16, 1.29]	11.90 ± 2.48; 12	12.64 ± 3.46; 12	t_(11)_ = −0.94, *p* = 0.366 [*p*_corr_ = 1]
# Correct						[2.5, 0.98]

CBD, cannabidiol; AMI, amisulpride; AVLT, auditory verbal learning test; d′, signal detection parameter; DRT, delayed response task, LNS, letter number sequencing; ROFT, Rey-Osterrieth complex figure test; SOPT, subject ordered pointing task; TMT-A, trail-making test A; TMT-B, trail-making test B; VBM, visual backward masking. Descriptive statistics, and paired t-test results (intention-to-treat set). Improvements are indicated by negative t-values except for SOPT (#error), DRT (Euclidian distance), TMT-B, and ratio TMT-B/TMT-A (both time in s).

**TABLE 3:** Effect size (Cohen’s d) for changes in neurocognitive test scores (t_0_-t_1_) and independent t-test results, to assess the equality of the effect sizes. Improvements are indicated by negative effect sizes except for SOPT (#error), DRT (Euclidian distance), TMT-B, and ratio TMT-B/TMT-A (both times in s).

	Cohen’s d		
	AMI d (t_0_ vs. t_1_) [95% CI]	CBD d (t_0_ vs. t_1_) [95% CI]	AMI vs. CBD (t_0_-t_1_) t_(df)_, p, d [95% CI]
VBM %hits	−0.59 [−1.23, 0.05]	−0.37 [−0.90, 0.15]	t_(27)_ = −0.30, *p* = 0.770 [p_corr_ = 1], d = −0.11 [−0.84, 0.62]
CPT d’ figures	−0.55 [−1.11, 0.02]	−0.23 [−0.60, 0.13]	t_(26)_ = −0.19, *p* = 0.850 [p_corr_ = 1], d = −0.07 [−0.81, 0.67]
CPT d’ symbols	−0.52 [−1.02, −0.02]	−0.47 [−0.83, −0.11]	t_(26)_ = −0.10, *p* = 0.923 [p_corr_ = 1], d = 0.04 [−0.71, 0.78]
LNS # correct	−0.67 [−1.72, −0.17]	−0.08 [−0.40, 0.55]	t_(22)_ = −1.87, *p* = 0.074 [p_corr_ = 1], d = −0.77 [−1.60, 0.07]
SOPT # errors	0.53 [0.06, 1.01]	0.03 [−0.64, 0.71]	t_(22)_ = 1.17, *p* = 0.255 [p_corr_ = 1], d = 0.48 [−0.34, 1.28]
DRT mean Euclidian distance	−0.81 [−1.58, −0.03]	0.16 [−0.36, 0.68]	t_(27)_ = −1.88, *p* = 0.071 [p_corr_ = 1], d = −0.70 [−1.45, 0.06]
AVLT immediate recall # correct	−0.33 [−0.79, 0.11]	−0.04 [−0.56, 0.48]	t_(27)_ = −0.85, *p* = 0.406 [p_corr_ = 1], d = −0.31 [−1.04, 0.42]
AVLT delayed recall # correct	−0.29 [−0.77, 0.19]	0.03 [−0.41, 0.48]	t_(27)_ = −0.96 *p* = 0.347 [p_corr_ = 1], d = −0.36 [−1.09, 0.38]
ROFT delayed recall standardized score	−0.63 [−1.14, −0.12]	−0.49 [−0.88, −0.10]	t_(25)_ = −0.89 *p* = 0.383 [p_corr_ = 1], d = −0.34 [−0.54, 1.08]
Digit-symbol coding # correct	−0.57 [−1.01, −0.13]	−0.41 [−0.68, −0.14]	t_(22)_ = 0.67 *p* = 0.511[p_corr_ = 1], d = 0.27 [−0.71, 0.78]
TMT-B time in s	0.63 [−0.08, 1.34]	0.32 [0.08, 0.56]	t_(22)_ = 0.91 *p* = 0.371 [p_corr_ = 1], d = 0.37 [−0.44, 1.18]
Ratio TMT-B/TMT-A time in s	0.21 [−0.56, 0.98]	0.11 [−0.51, 0.73]	t_(22)_ = 0.45 *p* = 0.655 [p_corr_ = 1], d = 0.19 [−0.62, 0.99]
Verbal fluency # correct	−0.34 [−0.89, 0.21]	−0.24 [−0.73, 0.26]	t_(22)_ = −0.15 *p* = 0.880 [p_corr_ = 1], d = −0.06 [−0.86, 0.74]

CBD, cannabidiol; AMI, amisulpride; AVLT, auditory verbal learning test; d′, signal detection parameter; CPT, continous performance task; DRT, delayed response task, LNS, letter number sequencing; ROFT, Rey-Osterrieth complex figure test; SOPT, subject ordered pointing task; TMT-A, trail-making test A; TMT-B, trail-making test B; VBM, visual backward masking.

Changes in neurocognitive performance were not systematically correlated with psychopathological improvements (PANSS total, general, positive, and negative symptom scores). Only 4 out of 104 (13 neuropsychological and 4 psychopathological parameter per treatment group) correlation coefficients between difference scores became “significant” (note, that about 5 (i.e., 5%) coefficients can be expected by chance alone): In the AMI group, the Letter Number Sequencing performance correlated with PANSS total (r = -0.69; *p* = 0.026 [p_corr_ = 1], N = 10), and PANSS general scores (r = -0.81; *p* = 0.005 [p_corr_ = 0.600], N = 10), while in the CBD group the verbal fluency performance was significantly associated with PANSS total (r = -0.65; *p* = 0.024 [p_corr_ = 1], N = 12) and PANSS general (r = -0.61; *p* = 0.037 [p_corr_ = 1], N = 12). The association with PANSS positive did not reach significance (r = -0.52; *p* = 0.085 [p_corr_ = 1], N = 12).

In addition, changes in neurocognitive performance were also not systematically associated with changes in serum anandamide levels (Table 4). Solely improvements on the Continuous Performance Test–figure stimuli (sustained attention, median slope = 0.99, 95% CI [0.03, 4.21]; *p* = 0.046 [p_corr_ = 1]) and the Digit-Symbol Test (processing speed, median slope = 3.31, 95% CI [1.72, 8.95], *p* = 0.012 [p_corr_ = 1]) in the AMI and CBD group respectively, were associated with higher anandamide levels.

**TABLE 4:** Association of change in serum anandamide levels and change in neurocognitive performance. Wilcoxon signed-rank test of the null hypothesis that the distribution of slopes is symmetric about zero. Statistical significant changes (p ≤ 0.05) are indicated in bold.

	Amisulpride		CBD	
	Slope (mean ± SD; N)	*Z*, *P* [*P*_*corr*_]*, median slope* [95% CI]	Slope (mean ± SD; N)	*Z*, *P* [*P*_*corr*_]*, median slope* [95% CI]
VBM %hits	40.10 ± 123.77; 6	0.11, 0.917 [1], 2.94 [−30.87, 148.23]	15.36 ± 34.71; 11	1.16, 0.248 [1], 8.82 [−7.10, 42.98]
CPT d’ figures	1.72 ± 2.41; 6	1.99, **0.046** [1]**, 0.99 [0.03, 4.21]**	0.26 ± 1.66; 11	0.80, 0.424 [1], 0.19 [−0.81, 1.43]
CPT d’	1.71 ± 3.81; 6	1.15, 0.249 [1], 0.36 [−0.19, 4.98]	0.30 ± 2.02; 9	1.51, 0.131 [1], 0.24 [−0.21, 1.95]
LNS # correct	6.15 ± 12.33; 6	1.48, 0.138 [1], 1.85 [−0.58, 17.38]	−0.12 ± 2.51; 9	0.00, 1,000 [1], 0.14 [−2.20, 2.20]
SOPT # errors	−0.23 ± 3.29; 7	−0.37, 0.715 [1], 0.00 [−3.61, 3.45]	1.47 ± 2.70; 11	1.72, 0.086 [1], 1.57 [−0.63, 3.33]
DRT mean euclidian distance	−21.11 ± 86.48; 6	−0.31, 0.753 [1], −5.58 [−113.38, 53.76]	−15.10 ± 28.01; 11	−1.25, 0.213 [1], −9.89 [−37.10, 2.78]
AVLT immediate recall # correct	4.6 ± 11.84; 7	0.73, 0.465 [1], 0.00 [−4.23, 15.78]	0.53 ± 4.93; 12	−0.05, 0.959 [1], 0.00 [−1.56, 4.04]
AVLT delayed recall # correct	5.85 ± 14.78; 7	0.41, 0.686 [1], 0.00 [−5.24, 22.10]	−1.33 ± 4.85; 11	−1.07, 0.285 [1], −1.30 [−4.76, 1.65]
ROFT delayed recall standardized score	22.15 ± 47.90; 6	0.11, 0.917 [1], 14.41 [−11.07, 78.03]	−1.22 ± 15.39; 11	0.45, 0.657 [1], 1.28 [−9.81, 6.52]
Digit-symbol coding # correct	8.02 ± 17.04; 6	1.214, 0.225 [1], 3.61 [−3.45, 24.41]	4.30 ± 4.31; 9	2.52**, 0.012** [1]**, 3.31 [1.72, 8.95]**
TMT-B time in s	−6.73 ± 175.76; 5	−0.14, 0.893 [1], −4.19 [−286.24, 156.87]	−13.78 ± 30.78; 9	−1.60, 0.110 [1], −14.90 [−37.32, 14.04]
Ratio TMT-B/TMT−A time in s	0.43 ± 3.47; 5	0.41, 0.686 [1], 0.88 [−5.10, 3.99]	0.44 ± 2.94; 9	−0.53, 0.594 [1], −0.19 [−1.52, 3.67]
Verbal fluency # correct	−3.54 ± 12.32; 7	−0.11, 0.917 [1], 0.14 [−18.94, 6.71]	5.88 ± 14.20; 9	1.24, 0.214 [1], 1.26 [−0.30, 21.59 ]

AVLT, auditory verbal learning test; d′, signal detection parameter; DRT, delayed response task, LNS, letter number sequencing; P, *p*-value; ROFT, rey-osterrieth complex figure test; SOPT, subject ordered pointing task; TMT-A, trail-making test A; TMT-B, trail-making test B; VBM, visual backward masking; Z, Wilcoxon signed-rank standardized test statistic.

## Discussion

This study shows that both CBD and AMI improve neurocognitive functioning with comparable efficacy in young, acutely ill schizophrenia patients. Interestingly, AMI improved working and visual memory performance as well as the processing speed, while CBD treatment led to improvements in processing speed, visual memory, visuomotor coordination, and sustained attention. However, all effect sizes were similar for both treatment groups.

In consideration of previous studies showing limited (McGuire et al., 2018) to no (Boggs et al., 2018) effect of CBD on neurocognitive functioning as an add-on medication in sub-acutely and chronically ill schizophrenia patients, our results indicate that CBD may be more beneficial when administered during the earlier acute phases of the illness. Neuroprotective properties of CBD have been described earlier (Hampson et al., 1998), and more recent data suggests anti-inflammatory effects of CBD as well (Mori et al., 2017), potentially contributing to an effectiveness at earlier stages of the disease. In addition, the influence of an add-on treatment is not clear, and potential pharmacodynamic interactions need to be considered. McGuire et al. (McGuire et al., 2018) accepted a stable treatment with antipsychotics only and observed a trend in favor of CBD (at a dosage of 1 g per day) in improving cognitive symptoms as assessed by the Brief Assessment of Cognition in Schizophrenia (BACS), an instrument basically covering the cognitive domains assessed in our study. In the study of Boggs et al. (Boggs et al., 2018), in which no beneficial CBD effects were observed, the patients were allowed to take a much broader spectrum of concomitant medication, including antipsychotics, antidepressants, and mood stabilizers and combined administration of these drugs. In addition, the daily dosage of CBD was limited to 600 mg.

Interestingly, data on the effects of AMI on cognitive function in schizophrenia patients is limited to a few open-label studies and randomized controlled trials (Tyson et al., 2004; Wagner et al., 2005; Mortimer et al., 2007; Wang et al., 2008; Kumar and Chaudhury, 2014). Consistent with our findings, AMI ameliorated cognitive impairments in all studies. Furthermore, the reported effect size of 0.4 for the global cognitive index after an 8-weeks treatment (Wagner et al., 2005) is comparable to the median effect size of 0.53 observed in the current study after four weeks of AMI treatment.

It has been suggested that the combined serotonin (5-HT_2A_) and dopamine-2 (D_2_) receptor blockade of second-generation antipsychotics is relevant for their ameliorating effects on neurocognitive impairments (Wagner et al., 2005). However, AMI is a second-generation antipsychotic with almost no affinity to 5-HT_2A_ receptors but a high affinity to block dopamine-3 (D_3_) receptors (Schoemaker et al., 1997). Nevertheless, AMI reduces cognitive impairments with at least similar efficacy as high 5-HT_2A_ receptor affine second-generation antipsychotics such as olanzapine or risperidone (Tyson et al., 2004; Wagner et al., 2005; Tyson et al., 2006; Mortimer et al., 2007; Wang et al., 2008; Kumar and Chaudhury, 2014). Furthermore, significant improvements in attention (Wagner et al., 2005; Tyson et al., 2006), executive function (Wagner et al., 2005), and auditory verbal learning (Mortimer et al., 2007) were only observed in patients treated with AMI. These findings suggest that low or no 5-HT_2A_ affinity may be more beneficial for cognition than high affinity (Tyson et al., 2004; Wagner et al., 2005). Although AMI and other second-generation antipsychotics have a considerable affinity to D_3_ receptors, little is known about the role of D_3_ receptor antagonism in ameliorating positive, negative, and cognitive symptoms (Gross and Drescher, 2012; Sokoloff and Le Foll, 2017). In preclinical studies, D_3_ receptor antagonists improved cognitive function, emotional processing, executive function, flexibility, and social behavior, but the few clinical studies with compounds of high affinity for D_3_ receptors and different degrees of selectivity over D_2_ receptors give only limited insight into the therapeutic potential of selective D_3_ antagonism (Gross and Drescher, 2012; Sokoloff and Le Foll, 2017).

Although current antipsychotics can yield modest beneficial effects on neurocognitive functioning (McCleery and Nuechterlein, 2019), cognitive impairments continue to pose a burden on patients. Therefore, novel compounds with a different mechanism of action are currently investigated, such as cholinergic agents, dopamine D_1_ agonists, glutamatergic agents (Buchanan et al., 2007), and CBD (Boggs et al., 2018). CBD seems to mediate its antipsychotic effects by modulating the endocannabinoid system (Leweke et al., 2012; Rohleder et al., 2016; Leweke et al., 2018). More precisely, its antipsychotic actions have been found to be related to increased levels of the endocannabinoid anandamide (Leweke et al., 2012), e.g., by blocking the anandamide degrading enzyme fatty acid amide hydrolase (FAAH) (Leweke et al., 2012) or the fatty amide binding proteins (FABPs) that transport anandamide to the FAAH enzyme (Elmes et al., 2015). Elevated anandamide levels can, in turn, interact with other neurotransmitter (e.g., dopamine) systems via cannabinoid type 1 receptors (CB_1_R) (Giuffrida et al., 1999; Giuffrida et al., 2004; Leweke, 2012), enhance glucose metabolism via peroxisome proliferator-activated receptor-γ (PPARγ) (Bouaboula et al., 2005) or modulate the immune function via cannabinoid type 2 receptors (CB_2_R). Other suggested pharmacological effects of CBD include: the activation of the vanilloid receptor 1 (TRPV1, transient receptor potential cation channel subfamily V member 1) (Bisogno et al., 2001; De Petrocellis et al., 2011), negative allosteric modulation of CB_1_R (Laprairie et al., 2015; Sabatucci et al., 2018), the facilitation of serotonergic neurotransmission via allosteric 5-HT_1A_ receptor modulation (Rock et al., 2012; Hind et al., 2016; Sonego et al., 2016), and modulation of glucose homeostasis and inflammatory processes by PPARγ activation (Esposito et al., 2011; Hind et al., 2016; Sonego et al., 2018).

However, further research is needed to clarify which of these pharmacological mechanisms contribute to CBD’s beneficial effects on cognition in acutely ill patients. It may be that CBD reduces cognitive impairments by reducing the proposed synaptic dopaminergic excess indirectly via CB_1_R activation by anandamide (Giuffrida et al., 1999; Giuffrida et al., 2004; Leweke, 2012). In this study, the neurocognitive performance changes were not systematically associated with changes in serum anandamide levels, indicating that CBD’s effects on cognition are mediated via different mechanisms, in particular given the fact that in the same patients, the significant increase in serum anandamide levels has been shown to be significantly associated with clinical improvement (Leweke et al., 2012). It has been suggested that the stimulation of 5-HT_1A_ receptors may improve cognition in schizophrenia (Meltzer and Sumiyoshi, 2008). Thus, the allosteric 5-HT_1A_ receptor modulation by CBD may also be an additional relevant mechanism of action. This hypothesis is consistent with the preclinical finding that CBD attenuates cognitive impairments in a schizophrenia-like animal mode and that these effects can be blocked by a 5-HT_1A_ receptor antagonist but not by CB_1_ and CB_2_ receptor antagonists (Rodrigues da Silva et al., 2020). Thus, the allosteric 5-HT_1A_ receptor modulation by CBD may also be an additional relevant mechanism of action. Notably, none of these mechanisms alone or a combination of different mechanisms seem to be more effective than the D_2_/D_3_ receptor blockade by AMI, as the efficacy of CBD and AMI was comparable in our study.

Changes in neurocognitive performance were also not systematically correlated with psychopathological improvements. Only PANSS total and PANSS general score were associated with the performance in one of three working memory tests (Letter Number Sequencing) in the AMI group and with the verbal learning performance in the CBD group. This finding supports the view that cognitive deficits are not merely the result of other schizophrenia symptoms (Keefe et al., 2006). In particular, the independence of cognitive deficits from positive symptoms has been shown previously (Green, 1996; Mohamed et al., 1999; Hughes et al., 2003; Gladsjo et al., 2004; Wagner et al., 2005; Keefe et al., 2006). On the other hand, a relationship between negative symptom severity and cognitive performance has been observed (Mohamed et al., 1999; Gladsjo et al., 2004; Keefe et al., 2006; Üçok et al., 2020). Furthermore, improvements in negative symptoms have been found to be associated with amelioration of cognitive deficits in people with schizophrenia treated with olanzapine or AMI for eight weeks (Wagner et al., 2005). The authors suggested that the same mechanisms may partly mediate both improvements in negative symptoms and cognitive performance. However, this hypothesis is not supported by our data. It may be that the treatment duration was not long enough to detect an association in this group. Furthermore, as discussed above, our findings indicate that CBD affects psychopathology via an anandamide-dependent pathway (Leweke et al., 2012), while its effects on cognitive performance seem to be mediated by another mechanism. This hypothesis is supported by the absence of a systematic correlation between the changes in neurocognitive performance and psychopathological improvements.

### Strengths and Limitations

The major strength of our study is the monotherapeutic, parallel-group design (Leweke et al., 2012). In contrast to previous add-on studies (Boggs et al., 2018; McGuire et al., 2018), our study design allows for investigating the therapeutic potential of the substance more precisely as no pharmacodynamic interactions need to be considered when assessing the effects of CBD. However, the lack of a placebo condition does not allow for an estimate of a potential placebo response to cognitive functioning. Nevertheless, these tests are generally considered robust for rater bias because of their objective character. While placebo-effects in cognitive trials in schizophrenia have been considered fairly small (Keefe et al., 2017), we cannot rule out practice effects on the improvements. Parallel versions of each cognitive test were used at t_0_ and t_1_, and all tests have been reported to have a good test-retest reliability and low item-specific learning. While we acknowledge the potential development of test-taking strategies and/or procedural learning as potential confounders in our trial, their contribution to the observed improvement in cognitive scores after four weeks of treatment is likely quite limited (Goldberg et al., 2010).

Further, the administration of lorazepam as a co-medication (up to 7.5 mg per day) may have influenced results as initial use of lorazepam was higher in the AMI than in the CBD group (Leweke et al., 2012) with a similar mean dosage at the end of the trial. This may have caused a cognitive improvement due to reduction in lorazepam favoring the AMI group, although the effect of short-term benzodiazepines on cognitive performance in schizophrenia is not well investigated (Baandrup et al., 2017; Fond et al., 2018). Furthermore, we included only acutely ill patients in earlier phases of the disease with a mean age of 29.7 ± 8.3 and 30.6 ± 9.4 years in the CBD and AMI group, respectively. In this group of patients, CBD seems to be more effective than in older sub-acutely (40.9 ± 12.5 years (McGuire et al., 2018)) and chronically (48.4 ± 9.3 years (Boggs et al., 2018)) ill patients. However, further studies investigating CBD’s therapeutic effects in first-episode psychosis or the prodromal phase are needed to confirm this hypothesis and investigate the contributing factors.

The current study is limited by the small sample size and comparatively short treatment duration. The actual sample size of *n* = 14–15 per treatment group is sufficient to detect large effect sizes (i.e., Cohen’s d) of 1.1 with a power of 80% at two-sided alpha 5%. However, the calculated 95% confidence intervals still give useful ranges for true differences that are compatible with the data obtained. Likewise, differences outside the intervals are not compatible with the data. Furthermore, as above mentioned, we did not make any multiplicity adjustments due to the exploratory character of the study, and none of the *p*-values would be significant after Bonferroni correction. Consequently, our exploratory finding that CBD improves neurocognitive functioning needs to be confirmed in a larger cohort of acutely ill schizophrenia patients (i.e., in a large RCT with neurocognitive functioning as primary objective). Furthermore, it needs to be investigated whether the maximal effect of CBD had already been achieved after four weeks of treatment or whether an extended treatment duration will lead to larger effects.

## Conclusion

This exploratory study shows that both CBD and AMI improve neurocognitive functioning with comparable efficacy in younger and acutely ill schizophrenia patients. However, larger RCTs are needed to confirm this explorative finding. Furthermore, our data indicates that CBD may affect psychopathology and cognitive performance via different physiological mechanisms. While improvements in psychopathology were significantly associated with an increase in serum anandamide levels (Leweke et al., 2012), cognitive improvements (if at all present) seemed to be induced via anandamide-independent pathways. Several alternative mechanisms of action have already been suggested for CBD, including an allosteric 5-HT_1A_ receptor modulation that may be relevant for CBD’s effects on neurocognitive functioning. However, the actual involvement of 5-HT_1A_ receptor modulation and other postulated mechanisms of action need to be examined explicitly in future studies.

## Data Availability

The datasets presented in this article are not readily available because they are available only to approved collaborators and competent authorities. Requests to access the datasets should be directed to markus.leweke@zi-mannheim.de.
